# Structural explanations for inequality reduce children’s biases and promote rectification only if they implicate the high-status group

**DOI:** 10.1073/pnas.2310573120

**Published:** 2023-08-21

**Authors:** Rachel A. Leshin, Marjorie Rhodes

**Affiliations:** ^a^Department of Psychology, New York University, New York, NY 10003

**Keywords:** inequality, development, bias, intervention

## Abstract

Children begin to participate in systems of inequality from a young age, demonstrating biases for high-status groups and willingly accepting group disparities. For adults, highlighting the *structural* causes of inequality (i.e., policies, norms) can facilitate adaptive outcomes—including reduced biases and greater efforts to rectify inequality—but such efforts have had limited success with children. Here, we considered the possibility that, to be effective in childhood, structural interventions must explicitly address the role of the high-status group in creating the unequal structures. We tested this intervention with children relative to a) a structural explanation that cited a neutral third party as the creator and b) a control explanation (*N* = 206, ages 5 to 10 y). Relative to those in the other two conditions, children who heard a structural explanation that cited the high-status group as the structures’ creators showed lower levels of bias, perceived the hierarchy as less fair, and allocated resources to the low-status group more often. These findings suggest that structural explanations can be effective in childhood, but only if they implicate the high-status group as the structures’ creators.

Children become aware of group-based hierarchies from a young age (1) and quickly become active participants in them: Children often favor those from high-status groups (2) and willingly accept (3)—and even perpetuate (4)—group inequalities. Among adults, highlighting the structural causes of inequality (5) encourages actions to rectify such disparities (6) and predicts lower levels of bias toward low-status groups (7). Might teaching young children about the structural roots of inequality yield similar benefits, facilitating an adaptive response to and understanding of inequality from early in development?

Correlational research is consistent with this possibility: Children who endorse structural explanations for economic inequalities develop less racial bias against Black Americans than those who do not (8). Experimental evidence of a causal link between structural explanations and children’s attitudes, beliefs, and behaviors, however, is mixed. When structural explanations are taught to children, they can understand them—children who learn that boys excel over girls at a new game due to differential access to resources (i.e., a structural rather than inherent cause), for example, expect such differences to remain stable as long as the structural constraint persists but not once it is revised (9, 10). Yet, such explanations often do little to change how children think and feel about the low-status group: These explanations do not appear to reduce children’s biases in favor of the group with more (11) nor do they consistently lead to rectification [(12); although they sometimes make children less accepting of the status quo, (13)]. Also limiting the potential of prior experimental work to inform interventions to reduce bias and promote rectification, most past experiments have exposed children to structural processes with obvious perceptual causes and effects [e.g., boys’ classrooms having more equipment for a new game than girls’ classrooms; (9, 10)], rather than ones arising from more abstract and complex systems (e.g., policies, norms, and/or laws, many of which date back generations). Thus, children’s response to and understanding of structural explanations that more closely reflect group-based inequalities observed in daily life remain unknown.

Additionally, there are several features of early social reasoning that may make the possible benefits of structural explanation interventions difficult to realize in early childhood, including the tendency to believe that what is true is right (14). As a result, when children are taught about the structural conditions that lead to inequality (as in refs. 10–13), they may assume that these conditions reflect how things should be and thus fail to question or challenge them. But while these social–cognitive biases present possible challenges, they might be mitigated if children learn, concretely, about the structures’ origins: specifically, the role of the high-status group in creating, maintaining, and perpetuating the structures that give rise to the inequality. By assigning selfish motives to the structures’ creators, such explanations have the potential to disrupt the intuitive link between what is true and what is right that may otherwise impede the benefits of structural interventions in childhood. This dimension has been overlooked in prior developmental work, with structural causes instead described in passive or ambiguous terms [e.g., “because of things that happened a long time ago”; (8, 13)] and the high-status group mentioned only as the structures’ beneficiaries (rather than their creators).

Here, we tested the efficacy of such an intervention relative to a) a structural explanation that names a third-party group as the creator (rather than the group in power) and b) a control. We first presented children with an inequality that was intended to mirror dynamics observed in everyday life. Then, we provided children with one of three explanations: a structural explanation that attributed the inequality to the high-status group (condition: High-Status Power), a structural explanation that attributed the inequality to a third-party (condition: Third-Party Power), or an explanation that did not appeal to structural causes at all (condition: Control; Fig. 1). Following the manipulation, we assessed children’s attitudes, beliefs, and behaviors toward the low-status group and their understanding of the inequality. To maintain consistency across conditions, dependent measures were presented in a fixed order that we thought would be easiest for children to follow. We recruited 206 elementary-school-age children (predominately from the U.S.; *M* = 7.56 y, *SD* = 1.65, range: 5.08–10.80; 50% girls, 50% boys; 54% White, 20% Asian, 17% multiracial, 5% Hispanic, 2% Black, 1% unreported) to participate (15).

**Fig. 1.**
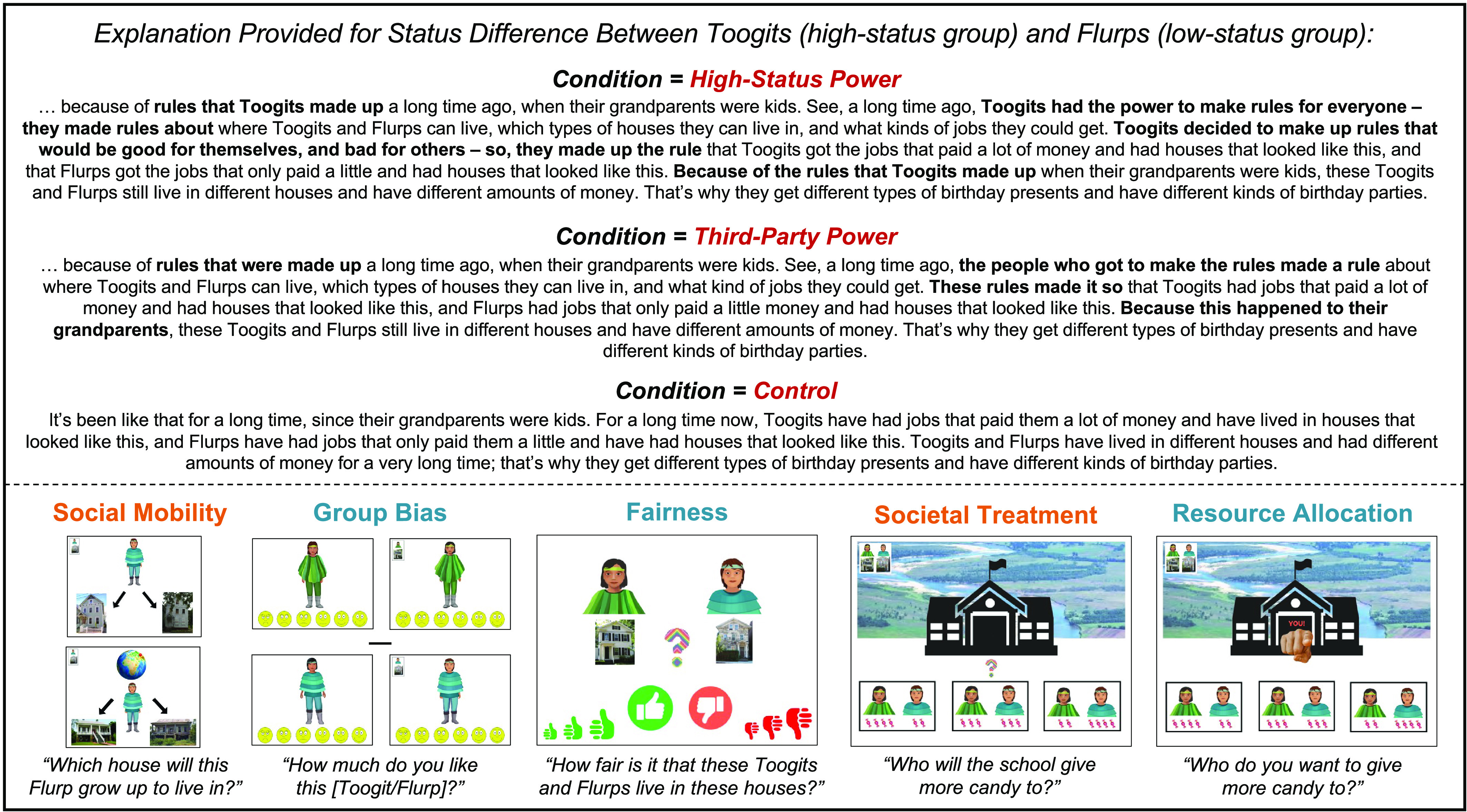
*Top*: Condition manipulation; *Bottom*: Dependent measures, displayed in order of presentation. Measures in blue probed responding*;* measures in orange probed understanding.

## Results

### Children’s Responses to the Inequality.

**Fig. 2.**
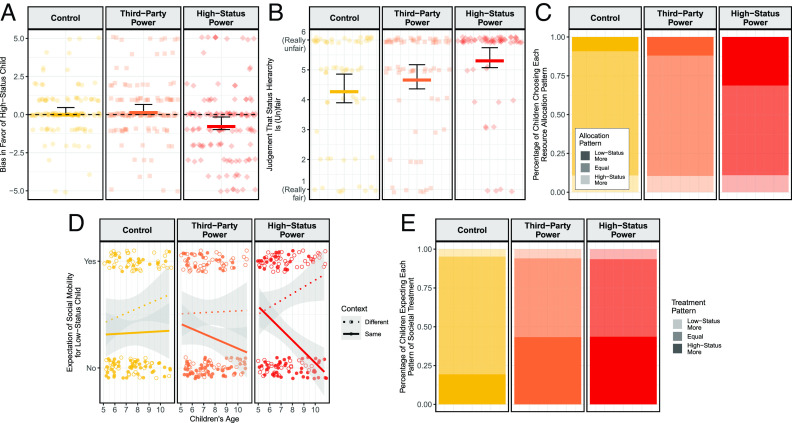
Children’s responses to (*Top*) and understanding of (*Bottom*) the inequality. Points are individual responses, horizontal lines are group means, lines are best-fitting regression lines, and error bars/bands are 95% CIs. (*A*) Group bias; (*B*) (un)fairness; (*C*) resource allocation; (*D*) social mobility; and (*E*) societal treatment.

Children in the High-Status Power condition showed more positive attitudes, beliefs, and behaviors toward the low-status group than those in the other two conditions. Compared to the Third-Party Power and Control conditions, children in the High-Status Power condition displayed less bias in favor of high-status children (main effect of condition *X*^2^(2) = 11.08, *P* = 0.004; contrast *p*s < 0.030), perceived the status hierarchy to be more unfair (main effect of condition *X*^2^(2) = 21.37, *P* < 0.001; contrast *p*s < 0.004), and allocated more resources to the low-status group [main effect of condition *X*^2^(2) = 12.32, *P* = 0.002; contrast *p*s < 0.030; overall, however, children tended to allocate equally, consistent with past research, (16); Fig. 2]. Strikingly, children in the High-Status Power condition expressed bias in favor of the *low-status* group (*M* = −0.57; *t*(129) = −2.74, *P* = 0.007), while children in the Third-Party Power condition showed bias in favor of the high-status group (*M* = 0.35; *t*(140) = 2.18, *P* = 0.031), and those in the Control condition showed no bias in either direction (*M* = 0.20; *t*(126) = 1.44, *P* = 0.153). In summary, structural explanations that implicated the high-status group yielded distinctive benefits for children’s responding to the low-status group.

### Children’s Understanding of the Inequality.

Children in the High-Status Power condition responded in this manner even though children in all conditions understood the inequality as intended. Indeed, across conditions, children expected the low-status child to have little social mobility throughout their lifespan if they remained in the same context but more social mobility if they moved to a new one, where the relevant structures need not apply (main effect of context *X*^2^(1) = 16.77, *P* < 0.001). This differentiation strengthened with age (age*context *X*^2^(1) = 8.86, *P* = 0.003), and particularly so in the High-Status Power condition (age*context*condition *X*^2^(2) = 5.93, *P* = 0.052; Fig. 2)—suggesting the development of a yet more sophisticated understanding among those who attributed the structures to the high-status group.

Children also understood the structural mechanisms as perpetuating present-day inequalities in both structural conditions. When asked whether a societal institution (a school) would favor the low-status group, favor the high-status group, or treat them equally, children in the High-Status Power and Third-Party Power conditions were more likely to expect biased treatment in favor of the high-status group relative to the Control condition (main effect of condition *X*^2^(2) = 11.08, *P* = 0.004; contrast *p*s < 0.020; Fig. 2).

### Robustness Check.

Because children’s social reasoning is sensitive to their environments, we conducted exploratory analyses to adjust for relevant environmental variables (e.g., parents’ political ideology, neighborhood poverty rate). All main effects of condition held (*p*s < 0.020).

## Discussion

Our findings suggest that structural explanations that identify the high-status group as the structures’ creators lead children to possess less bias toward low-status groups, perceive hierarchies as more unfair, and engage in more rectification. This was the case even though children understood the structural inequality without this added element, as demonstrated by their beliefs about social mobility and societal treatment (although structural explanations that implicated the high-status group supported a more robust understanding, with age, of the intractability of inequality without the possibility of structural change). To further assess the utility of this intervention, future research should test the efficacy of similar explanations when children themselves are embedded into the experimental paradigm (i.e., as members of the low- or high-status groups, similar to ref. 16), rather than mere observers of it. Our findings pave the way for these and other interventions, illuminating a critical component of structural explanations—the role and intentions of the high-status group—that may aid in the formation of more adaptive beliefs, attitudes, and behaviors across the lifespan.

## Materials and Methods

Methods and analyses were preregistered at https://osf.io/ersgq/ (for deviations, see “Deviations from Pre-registration” at https://osf.io/jd2p6/). The Institutional Review Board of New York University (IRB-FY2016-760) approved all consent procedures and protocols. Data, analysis scripts, and materials can be accessed at https://osf.io/jd2p6/. For more details, see *SI Appendix*.

## Supplementary Material

Appendix 01 (PDF)

## Data Availability

Anonymized data, analysis scripts, and materials have been deposited in Open Science Framework (https://doi.org/10.17605/OSF.IO/JD2P6) (15).
