# Supplementary material for: Structural explanations for inequality reduce children’s biases and promote rectification only if they implicate the high-status group
Source: Proc Natl Acad Sci U S A. 2023 Aug 21;120(35):e2310573120. doi: 10.1073/pnas.2310573120 (PMC10466091; doi:10.1073/pnas.2310573120)
Supplement: Supplementary file 1 — Appendix 01 (PDF) [file pnas.2310573120.sapp.pdf]

Supporting Information for

Structural explanations for inequality reduce children's biases and promote rectification only if they implicate the high-status group

Rachel A. Leshin[1] and Marjorie Rhodes[1]

[1]New York University, 6 Washington Place, New York, NY 10003

Corresponding Author: Rachel A. Leshin

Email: rachel.leshin@nyu.edu

This PDF file includes:

Supporting text

Supporting Information Text

Extended Methods

Participants. Children between 5 and 10 years were recruited via a remote and unmoderated platform for developmental research (Rhodes et al., 2020), which captures survey data and records webcam video. We aimed to recruit 189 children, as per an a priori power analysis conducted in G*Power (Faul et al., 2007) anchored on an effect we intended to replicate from a pilot study (i.e., the effect of structural explanations relative to essentialist explanations on children's perceptions of social mobility in a different context; *OR* = 2.30). Since exclusions occur at a rate of approximately 10% on our online platform, we pre-registered a plan to recruit 205 participants total. Due to the nature of our online platform, we ended up recruiting 221 children; of these, 206 children were screened as valid.

Our sample came from all across the U.S. (including 28 U.S. states; 7 participants were from Canada) and spanned the full age-range. Details of the gender and racial-ethnic breakdown are provided in the main text. Parents of participating children spanned the political ideology spectrum (from 1=Very Liberal to 7=Very Conservative; *M*=3.47, *SD*=1.50, *range*: 1-7) and varied with respect to their own beliefs about the causes of inequality (on a scale of 1=Strongly Disagree to 7=Strongly Agree, personal reasons *M*=4.11, *SD*=1.62, *range*: 1-7; societal reasons *M*=5.17, *SD*=1.32, *range*: 1-7; on binary measure, 65% endorsed societal reasons). Children and parents lived in neighborhoods with substantial variation with respect to levels of college education (*M*=41%, *SD*=10%, *range*: 13-67%), median rent (*M*=$1221.37, *SD*=$320.26, *range*: $581.10-2063.00), rate of poverty (*M*=.15, *SD*=.05, *range*: .05-.35), and income inequality (Gini Index score, wherein 0=full equality and 1=full inequality: *M*=.45, *SD*=.06, *range*: .33-.62).

Procedure. Children completed the study remotely from their home computers. Parents were briefed on the study content and encouraged to refrain from providing feedback or other cues to children during the study. After obtaining consent from parents and assent from children, we asked children to indicate their gender (*boy*, *girl, other or prefer not to say*), and the study began.

Introduction. Children were first introduced to two gender-matched kids (those whose gender was identified as "Other or prefer not to say" were shown images of girls). One kid was described as part of the *Toogit* group (and presented alongside other Toogits), and the other was described as a part of the *Flurp* group (and presented alongside other Flurps). Groups were visually differentiated from one another by the color and style of clothing (all stimuli were previously normed on relevant dimensions; see below for more details). Children were told that Toogits and Flurps live in the same town but are very different from one another.

Next, children learned the key difference between the two groups: status. To illustrate this difference, we described the resources and opportunities afforded to each group. Children saw the high-status house that the Toogit lives in and the low-status house that the Flurp lives in; they were told that grown-up Toogits have high-paying jobs and that grown-up Flurps have low-paying jobs; and they were told that, due to the different amounts of money that these grown-ups had, Toogit kids got fancy birthday gifts and hosted extravagant birthday parties, while Flurp kids got simple presents and did not get to have birthday parties at all.

Condition Manipulation**.** Next, children learned *why* it was that the Toogit and Flurp they saw, and their groups, were so different from one another. Children were randomly assigned to one of three conditions: High-Status Power, Third-Party Power, or Control (see Figure 1 in the main text). Children in the High-Status Power and Third-Party Power conditions completed a brief comprehension check, in which they indicated who got to make the rules about where Toogits and Flurps could live (options: Toogits, Flurps, or someone else). Most children (83%) responded correctly, and accuracy levels were similar across conditions. Those who responded incorrectly heard the explanation repeated and completed the comprehension check a second time; 71% responded correctly on this attempt, and those who did not (3% of the total sample) received corrective feedback. Next, children completed a series of measures assessing their *responses to* and *understanding of* the inequality, described below in the order in which they were presented.

Perceptions of Social Mobility (Understanding)**.** We first evaluated children's beliefs about the social mobility of the low-status child (i.e., the Flurp) across two contexts. The first was intended to mirror the present reality (i.e., where the conditions described in the story persisted). For this question, we asked children to consider what they had learned about the low-status child

and then indicate whether they thought that s/he would grow up to live in a high-status (coded as 1) or a low-status (coded as 0) house. The second context was a "different part of the world" (i.e., where the conditions described in the story could ostensibly change). To introduce this context, we first asked children to imagine a part of the world where all sorts of things were different and then asked two comprehension questions to confirm that children had imbued this context with the appropriate level of flexibility and constraint (i.e., whether people's favorite food there might be spinach, whether people there might have wings and fly). Most children answered correctly (65% "yes" to the first, 89% "no" to the second), and those who did not received corrective feedback. We then told children to imagine that the low-status child moved to this different part of the world and asked whether—in this different context—the low-status child would grow up to live in a high-status (coded as 1) or a low-status (coded as 0) house.

Group Bias (Responding). We next assessed children's liking of the high-status and low-status children from the story, as well as one other member of each group. Children indicated their liking of each child on a scale from "really don't like" (coded as 1) to "really like" (coded as 6). We computed our measure of group bias by subtracting children's ratings of the low-status children from their ratings of the high-status children.

Judgements of Fairness (Responding). Next, we asked children to indicate whether it was fair or not fair that the high-status and low-status children lived in their respective houses and had different amounts of money. We followed up by asking children to express *how* fair or unfair it was (sort of, pretty, or really) and coded responses on a scale of 1 (really fair) to 6 (really unfair).

Expectations of Societal Treatment (Understanding). We then assessed children's expectations of societal treatment by telling them that the neighborhood school had 6 extra pieces of candy to give out. Children were asked to predict how the school would allocate them: preferentially to the high-status child, preferentially to the low-status child, or equally.

Resource Allocation Decisions (Responding). Finally, we examined children's own allocation decisions by telling them that *they* were in charge of giving out the 6 pieces of candy. Children were asked to decide how to allocate the resources: preferentially to the high-status child, preferentially to the low-status child, or equally.

Debrief. All children received a short debrief, in which they were told that the groups of kids they had learned about were made-up and that in real life, children from different types of groups live in all different kinds of houses (and for many different reasons).

Parent Measures. At the end of the study, we asked parents a series of questions related to their social beliefs, which we used in our robustness analyses. First, we probed parents' endorsement of two explanations for why families struggle to make ends meet: one that cites personal factors (e.g., work ethic) and one that cites societal forces (e.g., access to education; adapted from Rizzo et al., 2022). Parents then selected the explanation that they thought *best* explained why parents struggle to make ends meet. Finally, parents self-reported their political ideology on a scale from 1 (Very Liberal) to 7 (Very Conservative).

Neighborhood Measures. Using the zip codes provided to us by each family, we extracted participant-level data on several relevant neighborhood characteristics (Chetty et al., 2018; U.S. Census American Community Survey), which we incorporated into our robustness analyses. We focused on four features that we perceived to be relevant to children's beliefs about inequality: the Gini Index (i.e., a measure of overall income inequality), median rent, poverty rate, and percentage of college-educated residents.

Video Coding. To ensure data quality, a trained research assistant coded ~20% of all study videos (n=41) for external interference (e.g., from parents), 50% of which (n=21) were independently coded by a second trained research assistant for reliability. We found extremely low levels of interference (1.31% of critical trials), and reliability between the two coders was excellent (98.90% agreement). As per our pre-registration, we retained all data in analyses.

Analytic Strategy. To examine the effect of condition on our outcomes of interest, we conducted a series of regressions (linear, binomial, and ordinal) and chi-square analyses in *R* Version 4.2.1. Primary analyses include condition and mean-centered age as fixed predictors. We followed up on all regressions with the *Anova()* function and report results of Wald X^2 tests. In our robustness checks, we added relevant features of parents' beliefs and children's neighborhoods to our models as additional fixed predictors (with all continuous predictors mean-centered).

Stimuli Norming. Prior to data collection, all stimuli used to represent Toogits (i.e., the high-status group) and Flurps (i.e., the low-status group) were normed on a series of relevant dimensions by a sample of adults (N=42) recruited via the crowdsourcing platform Prolific. Paired sample t-tests indicated that adults perceived the Toogit and Flurp from the story to have equal levels of status, to be equally attractive and friendly, and to be roughly the same age (all ps>.095). Additionally, adults reported liking the Toogit and Flurp to the same degree (p=.464). Analyses of the unfamiliar Toogits and Flurps included in the group bias measure were similarly well-matched on these metrics (all ps>.075). Finally, adults rated the Toogit group and Flurp group as a whole—as represented by clusters of members from each group, displayed as part of the introduction—as equally high in status, attractiveness, and friendliness (ps>.100), and they reported no difference in their liking of the two groups (p=1.0).
